# A novel incision technique of a totally implanted venous access port in the upper arm for patients with breast cancer

**DOI:** 10.1186/s12957-023-03043-4

**Published:** 2023-05-27

**Authors:** Xue Song, Shengying Chen, Yan Dai, Yang Sun, Xiaojie Lin, Jiafa He, Rui Xu

**Affiliations:** Breast department, Guangdong Provincial Hospital Of Chinese Medicine, No.111 Dade Rd, Yuexiu District, Guangzhou, Guangdong Province 510120 China

**Keywords:** Incision, Totally implantable venous access ports, Breast cancer

## Abstract

**Background:**

A totally implanted venous access port (TIVAP) in the upper arm is a safe and cost-effective vascular access device and is widely used in breast cancer patients. Traditional tunnelling technique increases the operation time and has an unsatisfied cosmetic effect, so we explored the feasibility, cosmetic effect and complications of an upper arm port with a novel incision in this retrospective study.

**Methods:**

We reviewed 489 cases of totally implantable venous access port implantation in the upper arm with two types of incisions in our centre from 1 January 2018 to 30 January 2022. The patients were divided into two different incision groups including the puncture site incision group (*n* = 282) and the conventional tunnelling group (*n* = 207). The comparison of the results was collected between the two groups, and contributing factors were analyzed for major complications.

**Results:**

A total of 489 patients were successfully implanted with arm ports using the puncture site incision technique (*n* = 282, 57.7%) and conventional tunnelling technique (*n* = 207, 42.3%). The average operation time of the two types of incisions was 36.5 ± 15 min in the puncture site incision group and 55 ± 18.1 min in the tunnel needle group (*P* < 0.05). In terms of complications, 33 catheter-related complications occurred (6.4%), including 9 cases of infection, 15 cases of catheter-related thrombosis and 7 cases of skin exposure. Fourteen patients in the puncture site incision group developed complications compared with 17 in the traditional incision group. There were no significant differences between the two groups in terms of overall complication events (5.0% and 8.2%, *P* = 0.145) while the same result was found in each complication event. Weight, total cholesterol and diabetes were found to be associated with device-related infections in the univariate Cox proportional hazard regression models. Diabetes was found to be associated with device-related infections in multivariate analysis while hypertension was associated with thrombosis.

**Conclusions:**

The puncture site incision method is a novel technique with a better cosmetic appearance and less operation time than the traditional tunnelling technique, providing a comparable overall rate of complications. It offers a preferable choice for clinicians when dealing with different situations of patients. It is worthy of being used and promoted for patients requiring the totally implanted venous access port in the upper arm.

## Background

A totally implanted venous access port (TIVAP) is an effective and safe vascular access device and is widely used in breast cancer patients who receive chemotherapy infusion for systematic therapy [1, 2]. The most common site for the implantation of TIVAP is the anterior chest via the jugular vein or subclavian vein [3, 4]. Retrospective studies have shown that TIVAP implanted in the upper arm is a safe and cost-effective vascular access device and is widely employed in breast cancer patients due to the low pneumothorax rate and better aesthetic appearance [5–7]. Many studies have shown that the appearance of the port site scar does impact the perception of patients with breast cancer, and they would like to choose a less noticeable site, such as the upper arm, for port placement [8, 9]. Therefore, we chose to employ the upper arm port rather than the chest port for breast cancer patients in 2018, not only in consideration of safety but also for a good aesthetic appearance. Usually, a horizontal incision was made approximately 2–3 cm below the puncture site, and the port was connected to the venous catheter through a subcutaneous tunnel [10]. After the surgery, there are two scars left on the upper arm: one is the puncture port, and the other is the incision. In recent years, we have tried to explore an alternate scar approach that has less scarring and is more desirable for the patient. Based on this, we employed a novel incision technique, puncture site incision, to minimize the visibility and appearance of the port site scar. Furthermore, the injury and complications will be reduced, and the operation time will be shortened by this technique. Currently, there is no randomized trial comparing this novel incision with the conventional incision in patients with breast cancer.

The aim of the present study was to retrospectively evaluate the feasibility and complications, such as infection and thrombus of the upper arm port with traditional incision and puncture site incision in female patients with breast cancer.

## Methods

### Patients

In this retrospective study, we reviewed 489 patients with early breast cancer who were implanted in the upper arm port at Guangdong Provincial Hospital of Chinese Medicine (Guangzhou City, Guangdong Province, People’s Republic of China) from 1 April 2018 to 30 Jan 2022 for the administration of chemotherapy. The surgeon decided which technique to use according to the situation of the patient. Patients were excluded if they had poor arm vein (brachial or basilic) conditions and were unsuitable for arm implantation. The chemotherapy regimens commonly contained anthracyclines, taxanes and other chemotherapy drugs. The patients were followed up until the upper arm port was removed. Information on the patients and any complications, such as infection and venous thrombosis, was retrieved from their medical records.

### Patients consent

All the procedures followed were carried out in compliance with the ethical standards and with the principles of the Declaration of Helsinki. This study was approved by the ethics committee of the Guangdong Provincial Hospital of Chinese Medicine (ZE2022-219). The possible advantages and risks of the procedure were explained to all the patients before voluntary approval to participate in the study. The patients were informed of the procedure and complications and signed the informed consent form.

### Procedure

**Fig. 1:**
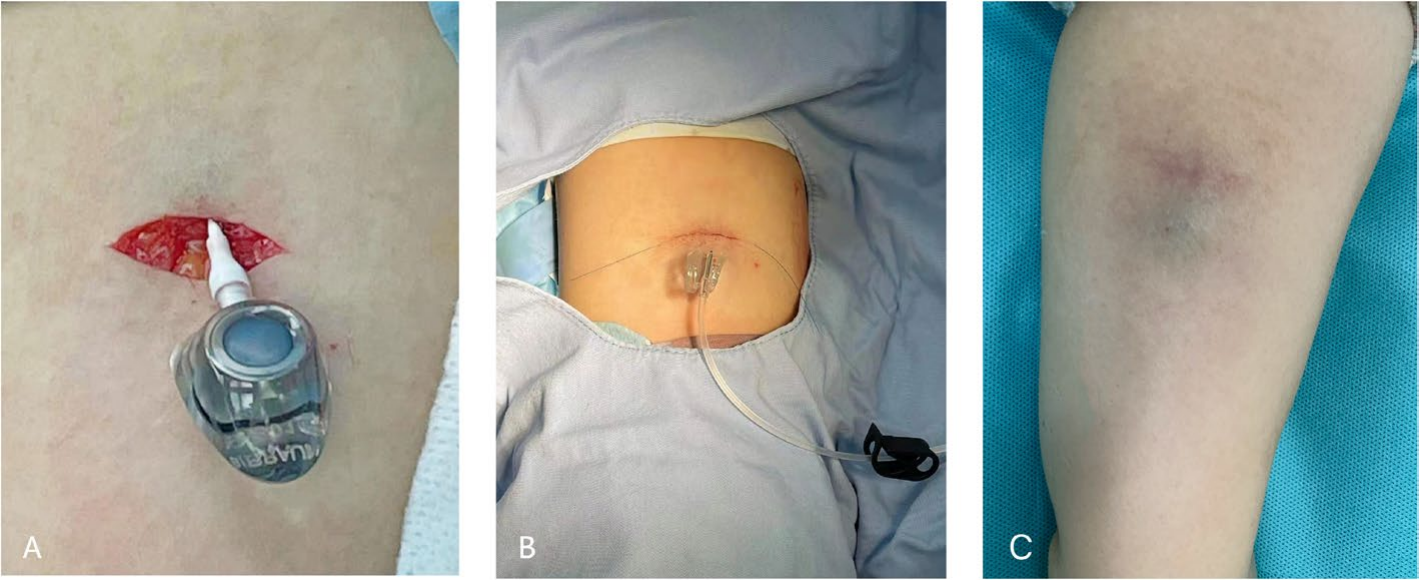
Puncture site incision without a subcutaneous tunnel (**A**). After suture (**B**). One scar remained (**C**)

All procedures were performed by senior surgeons using the same implantation protocol under local anaesthesia in an operation room with an X-ray machine and ultrasound device. All TIVAPs were implanted in the contralateral arm for unilateral breast cancer, whether or not lymphadenectomy was performed. The TIVAP was implanted on the side opposite to the lymphadenectomy or axillary metastases in cases of bilateral breast cancer. Since the port is smaller and appears to be more suited for subcutaneous implantation in the upper arm, we used a venous access port (CELSITE, BRAUN Medical) with a 5.0-French catheter size. The puncture site was chosen using ultrasonography while the patient was in the supine position with the target arm kept perpendicular to her body. It is based on the principle that a port can always be positioned in the arm, guaranteeing a catheter/vein ratio ≤ 1/3 (as most of the recent scientific literature suggests) or ≤ 0.45 (suggested by INS 2021 ed. Standards of Practice). Through proximal vein access (in the so-called green ZIM zone of Dawson) or in the yellow zone and subsequent tunnelling up to the passage between the yellow zone and the green zone, the basilic vein was the preferred choice for the puncture vessel in this study; however, when the basilic vein was difficult to identify or unsuitable for a puncture, the cephalic vein or brachial vein was employed. The major procedure was performed in the following manner. First, a puncture was performed, and a guidewire was inserted into the basilic vein (or brachial vein) under ultrasound guidance [11]. We used the ECG method and observed the change in the P wave. The P wave reaches its highest point when it reaches the cavoatrial junction (CAJ) to ensure that the tip is at the CAJ [12, 13]. Subcutaneous local anaesthetic (1% lidocaine) was administered to the puncture site and port area. Next, the surgeons made a horizontal incision through the puncture site, which we called the puncture site incision (Fig. 1A). A port pocket was created by separating the subcutaneous tissues, and a port hub was then implanted inside the pocket while being attached to the venous catheter (Fig. 1A). Finally, the incision was sutured with 4-0 absorbable sutures, and there was one scar left in the upper arm (Fig. 1B) with the novel incision technique. The healed wound and the scar are shown in Fig. 1C.

**Fig. 2:**
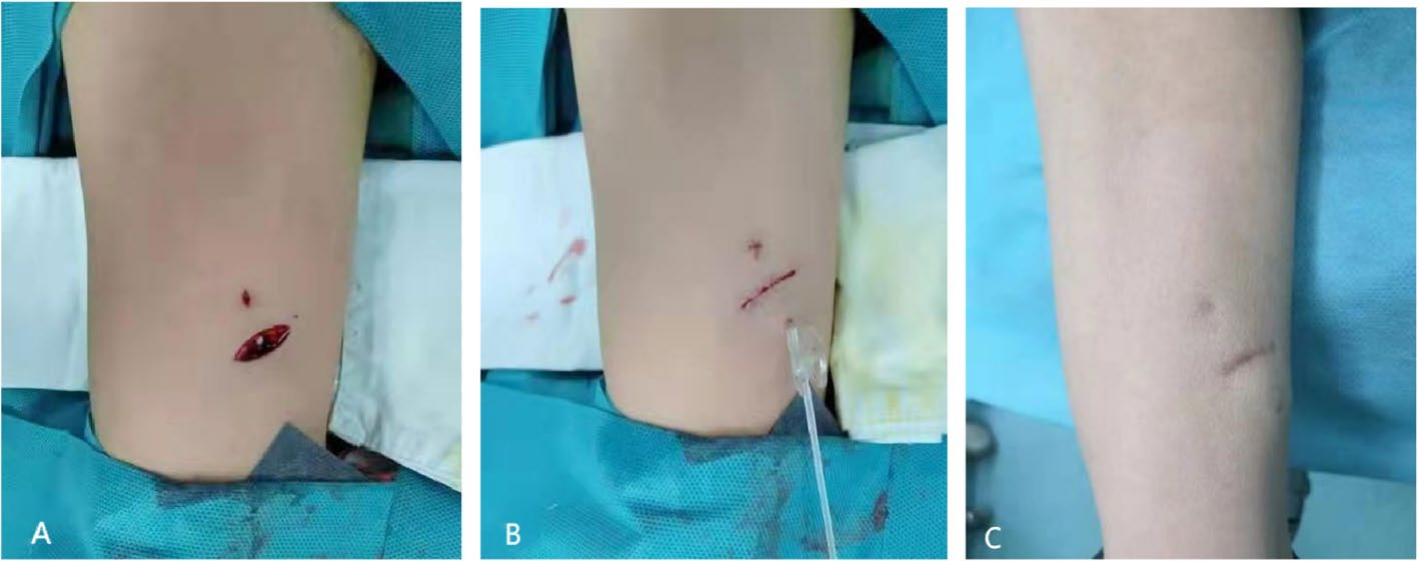
Traditional tunnel needle technique (the port hub was connected to the catheter through a subcutaneous tunnel) (**A**). After suture (**B**). Two scars are left (**C**)

A traditional incision was made approximately 2–3 cm below the puncture site [10]. A metal guide stick was used to create a subcutaneous tunnel, and a port hub was connected to the venous catheter through the tunnel. Finally, the port was implanted in the pocket (Fig. 2A). The two scars were left in the upper arm after healing [8] (Fig. 2B).

### Statistical analyses

We reviewed the medical records of the patients for information including their age, height, weight, BMI, hypertension, diabetes, incision type, coagulation parameters, lipid index, implantation site, implantation depth, operation time and breast cancer stage. The primary outcomes were operation time, cosmetic results and the occurrence of adverse events. The operation time was defined as the time from the vein puncture to suturing. The following conditions were used to define catheter and port infection: (1) when blood culture tests for microorganisms were positive, (2) or when blood culture tests were negative but when there was a high fever (temperature over 39 °C) with localized inflammation that included redness, heat and discomfort persisted in the port pocket [14, 15]. Venous thrombosis was confirmed by ultrasonography [16]. Skin exposure was defined as the port exposure to the skin.

The mean and standard deviation of descriptive variables were used in the statistical analysis, whereas counts and percentages were used to characterize the categorical variables. The *T* tests and chi-square tests were used to compare the parametric and nonparametric variables between the groups. To examine the risk factors for complications, we used univariate analysis with Pearson’s chi-square tests and *t* tests. In the multivariate analysis, factors with a *P* < 0.20 in the univariate analysis were selected, and a logistic regression model was used. *P* < 0.05 was used to denote statistical significance. SPSS (version 20, IBMC Corp., Armonk, NY, USA) was used for the statistical analysis.

## Result

The upper arm ports were successfully implanted in 489 patients, with 282 using a puncture site incision and 207 using the traditional tunnel needle technique. Table 1 summarizes the baseline characteristics and pathological features. The basilic vein was used for upper arm ports in 402 (82.2%) of the patients; for the remaining 87 patients, the brachial vein and cephalic vein were used because the basilic vein was unsuitable or puncture failure occurred. A comparison of the puncture site incision group and the traditional tunnel needle technique group revealed that the operation time of the traditional incision group was significantly longer (55 ± 18.1vs. 35.6 ± 15 min; *P* < 0.001).

**Table 1:** Patient characteristics (*n* = 487)

Characteristic	Puncture site incision technique (*n* = 282)	Traditional tunnel needle technique (*n* = 207)	*P*
Age (years)	50.7 ± 10.1	48 ± 10.0	0.004
BMI (kg/m^2^)	23.2 ± 3.51	22.8 ± 3.19	0.177
Hypertension	57 (20.4%)	28 (13.5%)	1
Diabetes	17(8.2%)	25 (8.9%)	1
PT (s)	11.5 ± 1.11	11.8 ± 1.19	0.001
APTT (s)	30.1 ± 5.67	32.0 ± 5.89	0.001
FIB (g/L)	3.25 ± 0.89	3.35 ± 0.88	0.213
ALB (g/L)	45.6 ± 3.35	44.7 ± 3.71	0.005
TG (mmol/L)	1.27 ± 0.82	1.37 ± 1.24	0.269
TC (mmol/L)	4.81 ± 0.96	4.90 ± 1.07	0.326
LDL-C (mmol/L)	3.09 ± 0.84	3.18 ± 1.01	0.258
TNM stage (*n*)			
I	39 (13.8%)	15 (7.2%)	1
II	112 (39.8%)	90 (43.5%)	
III	89 (31.6%)	74 (35.7%)	
IV	40 (14.9%)	30 (14.5%)	
Implantation site (*n*), access vein			
Basilic vein	266 (94.3%)	136 (65.7%)	1
Cephalic vein	0 (0%)	52 (25.1%)	
Brachial vein	16 (5.7%)	19 (9.2%)	
Implantation depth (cm)	36.7 ± 3.21	37.4 ± 2.88	0.015
Operation time (min)	35.6 ± 15	55 ± 18.1	< 0.001

*BMI* Body mass index, *TNM* Tumour–node–metastasis, *PT* Prothrombin time, *APTT* Activated partial thromboplastin time, *FIB* Fibrinogen, *ALB* Albumin, *TG* Triglyceride, *TC* Cholesterol, *LDL-C* Low-density lipoprotein

The TIVAP-related complications registered during the median time of 7.1 months of follow-up are summarized in Table 2. Fourteen patients in the puncture site incision group developed TIVAP-related complications compared with 17 in the traditional incision group (Table 2). In the novel technique group, the incidence of catheter-related infections, thrombosis and skin exposure was 2.5%, 1.8% and 0.7%, respectively, compared to 1.0%, 4.8% and 2.4% in the tunnelling group. There were no significant differences between the two groups in terms of overall complication occurrences (5.0% and 8.2%, *P* = 0.145). Several potential risk factors (age, BMI, hypertension, diabetes, incision type, coagulation parameters, lipid index, implantation site, implantation depth, operation time and stage of breast cancer) for the complications presented in Tables 3, 4, and 5 were analysed using a Cox regression model. There was no difference in the incidence of complications regardless of which vein (basilic, cephalic, brachial) was selected (Table 3).

**Table 2:** TIVAP-related complications

Complications	Puncture site incision technique	Traditional tunnel needle technique	*P*
Device-related infections	7 (2.5%)	2 (1.0%)	0.218
Catheter-associated venous thrombosis	5 (1.8%)	10 (4.8%)	0.052
Skin exposure	2 (0.7%)	5 (2.4%)	0.458
Total	14 (5.0%)	17 (8.2%)	0.145

**Table 3:** TIVAP-related complications with different puncture veins

Complications	Infections		Thrombosis		Skin exposure	
	Yes	No	Yes	No	Yes	No
Access vein						
Basilic vein	9 (2.2%)	393 (97.8%)	12 (3.2%)	390 (96.8%)	4 (1.0%)	398 (99%)
Cephalic vein	0 (0%)	52 (100%)	2 (1.9%)	50 (98.1%)	1 (1.9%)	51(98.1%)
Brachial vein	0 (0%)	35 (100%)	1 (3%)	34 (97%)	2 (6.1%)	33(93.9%)
* P*	0.38		0.88		0.69

**Table 4:** Univariate Cox proportional hazard regression analyses for device-related infections

	Device-related infections (*n* = 9)	No device-related infections (*n* = 480)	*P*
Age (years)	51.44 ± 12.48	49.49 ± 10.11	0.57
Height (cm)	159.78 ± 5.70	156.58 ± 5.53	0.09
Weight (kg)	63.50 ± 11.97	56.45 ± 8.63	0.02
BMI (kg/m^2^)	24.99 ± 5.00	23.02 ± 3.34	0.08
PT (s)	10.93 ± 0.74	11.71 ± 1.17	0.06
APTT (s)	27.39 ± 3.61	31.29 ± 5.87	0.07
FIB (g/L)	3.51 ± 0.74	3.31 ± 0.89	0.5
ALB (g/L)	43.27 ± 3.34	45.08 ± 3.59	0.13
TG (mmol/L)	1.31 ± 0.65	1.33 ± 1.09	0.95
TC (mmol/L)	4.19 ± 1.24	4.87 ± 1.01	0.04
LDL-C (mmol/L)	2.56 ± 1.25	3.16 ± 0.94	0.06
Implantation depth (cm)	37.56 ± 3.75	36.99 ± 3.09	0.58
Operation time (min)	45.56 ± 14.96	43.81 ± 19.06	0.78 0.22
Hypertension			
Yes	3 (33.3%)	82 (17.1%)	
No	6 (66.7%)	398 (82.9%)	
Diabetes			< 0.001
Yes	4 (44.4%)	38 (7.9%)	0.23
No	5 (55.6%)	442 (92.1%)	
Incision type			
Puncture site incision technique	7 (77.8%)	275 (57.3%)	
Traditional tunnel needle technique	2 (22.2%)	205 (42.7%)	
TNM stage (*n*)			
I	0 (0%)	54 (11.3%)	0.73
II	4 (44.4%)	198 (41.3%)	
III	2 (22.2%)	161 (33.5%)	
IV	3 (33.3%)	67 (14.0%)	
Access vein			
Basilic vein	9 (100%)	393 (81.9%)	0.38
Cephalic vein	0 (0%)	52 (10.8%)	
Brachial vein	0 (0%)	35 (7.3%)	

*BMI* Body mass index, *TNM* Tumour–node–metastasis, *PT* Prothrombin time, *APTT* Activated partial thromboplastin time, *FIB* Fibrinogen, *ALB* Albumin, *TG* triglyceride, *TC* Cholesterol, *LDL-C* Low-density lipoprotein

**Table 5:** Univariate Cox proportional hazard regression analyses for catheter-associated venous thrombosis

	Catheter-associated venous thrombosis (*n* = 15)	No catheter-associated venous thrombosis (*n* = 472)	*P*
Age (years)	49.5 ± 10.19	49.7 ± 9.52	0.94
Height (cm)	156.6 ± 5.57	156.3 ± 4.86	0.82
Weight (kg)	56.7 ± 8.79	53.7 ± 7.17	0.2
BMI (kg/m^2^)	23.1 ± 3.39	21.9 ± 2.74	0.76
PT (s)	11.6 ± 1.16	11.3 ± 1.33	0.2
APTT (s)	31.3 ± 5.85	28.4 ± 5.8	0.06
FIB (g/L)	3.31 ± 0.89	3.18 ± 0.89	0.58
ALB (g/L)	45.0 ± 3.62	45.3 ± 2.61	0.2
TG (mmol/L)	1.33 ± 1.09	1.28 ± 0.85	0.85
TC (mmol/L)	4.85 ± 1.02	4.92 ± 1.0	0.81
LDL-C (mmol/L)	3.14 ± 0.95	3.23 ± 0.8	0.73
Implantation depth (cm)	36.9 ± 3.09	38.4 ± 3.11	0.08
Operation time (min)	43.5 ± 18.9	52.9 ± 18.03	0.06 0.07
Hypertension			
Yes	15 (100%)	85(18%)	
No	0 (0%)	389(82%)	
Diabetes			0.51
Yes	2 (13.3%)	40 (8.5%)	0.06
No	13 (86.7%)	434 (91.5%)	
Incision type			
Puncture site incision technique	5 (33.3%)	277 (58.7%)	
Traditional tunnel needle technique	10 (66.6%)	197 (41.3%)	
TNM stage (*n*)			
I	5 (33.3%)	49 (10.4%)	0.32
II	5 (33.3%)	197 (41.7%)	
III	2 (13.3%)	161 (34.1%)	
IV	3 (20%)	67 (14.2%)	
Access vein			
Basilic vein	12 (80%)	390 (82.6%)	0.88
Cephalic vein	2 (13.3%)	50 (10.6%)	
Brachial vein	1 (6.7%)	34 (7.2%)	

*BMI* Body mass index, *TNM* Tumour–node–metastasis, *PT* Prothrombin time, *APTT* Activated partial thromboplastin time, *FIB* Fibrinogen, *ALB* Albumin, *TG* Triglyceride, *TC* Cholesterol, *LDL-C* Low-density lipoprotein

Device-related infections occurred in 7 patients in the puncture site incision group compared with 2 patients in the traditional incision group (Table 2). In the univariate Cox proportional hazard regression analyses, weight, TC and diabetes were significantly associated with device-related infections (Table 4). In the multiple logistic regression analysis, diabetes was found to be an independent risk factor for infection (*P* = 0.004) (Table 6).

**Table 6:** Univariate Cox proportional hazard regression analyses for skin exposure

	Skin exposure (*n* = 7)	No Skin exposure (*n* = 482)	*P*
Age (years)	51.71 ± 12.24	49.50 ± 10.13	0.57
Height (cm)	157.14 ± 6.41	156.64 ± 5.54	0.81
Weight (kg)	54.93 ± 8.72	56.60 ± 8.74	0.61
BMI (kg/m^2^)	22.14 ± 2.24	23.07 ± 3.39	0.47
PT (s)	11.60 ± 1.06	11.69 ± 1.17	0.83
APTT (s)	30.01 ± 4.32	31.24 ± 5.88	0.58
FIB (g/L)	3.75 ± 1.19	3.31 ± 0.89	0.19
ALB (g/L)	45.46 ± 5.70	45.04 ± 3.56	0.76
TG (mmol/L)	1.16 ± 0.61	1.33 ± 1.09	0.67
TC (mmol/L)	4.71 ± 0.87	4.86 ± 1.02	0.69
LDL-C (mmol/L)	3.10 ± 0.86	3.15 ± 0.95	0.88
Implantation depth (cm)	38.21 ± 3.49	36.98 ± 3.09	0.29
Operation time (min)	57.43 ± 23.89	43.64 ± 18.86	0.06 0.44
Hypertension			
Yes	2 (28.6%)	83 (17.2%)	
No	5 (71.4%)	399 (82.8%)	
Diabetes			0.59
Yes	1 (14.3%)	41 (8.5%)	0.14
No	6 (85.7%)	441 (91.5%)	
Incision type			
Puncture site incision technique	2 (28.6%)	280 (58.1%)	
Traditional tunnel needle technique	5 (71.4%)	202 (41.9%)	
TNM stage (*n*)			
I	2 (28.6%)	52 (10.8%)	0.44
II	1 (14.3%)	201 (41.7%)	
III	1 (14.3%)	162 (33.6%)	
IV	3 (42.9%)	67 (13.9%)	
Access vein			
Basilic vein	4 (57.1%)	398 (82.6%)	0.69
Cephalic vein	1 (14.3%)	51 (10.6%)	
Brachial vein	2 (28.6%)	33 (6.8%)	

*BMI* Body mass index, *TNM* Tumour–node–metastasis, *PT* Prothrombin time, *APTT* Activated partial thromboplastin time, *FIB* Fibrinogen, *ALB* Albumin, *TG* Triglyceride, *TC* Cholesterol, *LDL-C* Low-density lipoprotein

Catheter-related thrombosis occurred in 5 patients in the puncture site incision group compared with 10 patients in the traditional incision group (Table 2). All the patients underwent systemic anticoagulant therapy, and the TIVAPs remained in use without further complications. No clinical characteristics were found to increase the risk of thrombosis in the univariate Cox proportional hazard regression analyses (Table 5), while hypertension was found to be an independent risk factor for catheter-related thrombosis (*P* < 0.001) (Table 7).

**Table 7:** Multiple logistic regression analysis for device-related infections

	*B*	SE	Wald *X* ^2^	*P*	OR	OR 95%CI	
						Lower	Upper
Intercept	38.087	34.537	1.216	0.27			
Height	− 0.278	0.214	1.691	0.193	0.757	0.497	1.152
Weight	0.181	0.258	0.492	0.483	1.199	0.722	1.989
BMI	− 0.649	0.665	0.952	0.329	0.523	0.142	1.924
PT	0.133	0.268	0.247	0.62	1.142	0.676	1.931
APTT	0.18	0.109	2.712	0.1	1.197	0.966	1.484
ALB	0.133	0.095	1.955	0.162	1.142	0.948	1.377
TC	0.259	0.578	0.201	0.654	1.296	0.417	4.024
LDL-C	0.531	0.629	0.713	0.398	1.701	0.496	5.832
Diabetes	− 2.426	0.846	8.227	0.004	0.088	0.017	0.464

*BMI* Body mass index, *PT* Prothrombin time, *APTT* Activated partial thromboplastin time, *FIB* Fibrinogen, *ALB* Albumin, *TC* Cholesterol, *LDL-C* Low-density lipoprotein

Skin exposure occurred in 2 patients in the puncture site incision group compared with 5 patients in the traditional incision group (Table 2). All patients had the port removed. In the univariate Cox proportional hazard regression analyses, operation time was significantly associated with skin exposure (Table 8). In the multiple logistic regression analysis, no clinical characteristics were found to be an independent risk factor for infection (Table 9).

**Table 8:** Multiple logistic regression analysis for catheter-associated venous thrombosis

	*B*	SE	Wald *X* ^2^	*P*	OR	OR 95%CI	
						Lower	Upper
Intercept	7.836	4.015	3.809	0.051			
APTT	0.09	0.066	1.857	0.173	1.094	0.961	1.245
Implantation depth	− 0.162	0.088	3.402	0.065	0.85	0.716	1.01
Operation time	− 0.004	0.018	0.057	0.811	0.996	0.961	1.032
Hypertension	19.973	0	< 0.001	< 0.001	472,173,041.9	472,173,041.9	472,173,041.9
Incision type	− 0.61	0.66	0.853	0.356	0.543	0.149	1.983

*APTT* Activated partial thromboplastin time

**Table 9:** Multiple logistic regression analysis for skin exposure

	*B*	SE	Wald *X* ^2^	*P*	OR	OR 95%CI	
						Lower	Upper
Intercept	8.563	2.017	18.031	0			
Incision type	− 0.696	0.948	0.538	0.463	0.499	0.078	3.2
FIB	− 0.533	0.341	2.44	0.118	0.587	0.301	1.145
Operation time	− 0.027	0.019	2.036	0.154	0.973	0.937	1.01

*FIB* Fibrinogen

## Discussion

In recent years, many centres have used an upper arm venous port as an alternative to a chest port because of the lower puncture-related complications and a better aesthetic appearance for patients with breast cancer to complete intravenous chemotherapy [4–6]. The success of port insertion is a major concern for both surgeons and patients. However, little attention has been given to the effects of the scar and the operation time from the port procedure, which are frequently significant aspects of a patient’s treatment. Thus, we are attempting to investigate a unique incision that has less scarring and is more desirable for patients. To the best of our knowledge, this is the first study to evaluate the insertion types for upper arm port implantation.

In this study, we evaluated the feasibility, aesthetic appearance, operation time and safety of using two different incision techniques on upper arm TIVAPs in 489 patients with breast cancer. First, this novel technique has a short learning curve for surgeons in terms of both the operation time and the incidence of complications compared with the tunnelling technique in practice. With the novel insertion technique, we make one incision to implant the port, and there is only a port scar left in the upper inner arm rather than two conventional scars. We omit the procedure step of long-range subcutaneous drilling with a tunnel needle, for the reasons that these help to avoid injury and congestion of subcutaneous tissue and to help incision healing.

Furthermore, the operation time of the novel incision group was significantly shorter than that of the traditional incision group (Table 1), indicating that the novel incision will save procedure time. The patients with complications of skin exposure had longer operation times in this study. This could be related to spending time on constructing a subcutaneous tunnel and resulting in the injury of subcutaneous tissue. Nevertheless, a novel technique shortens the operation duration and reduces subcutaneous injury, lowering the risk of skin exposure after the surgery.

However, a good reason for tunnelling is the need for puncturing the vein in a very high position, close to the axilla, through proximal vein access (in the so-called yellow ZIM zone of Dawson) and subsequent tunnelling up to the passage between the yellow zone and the green zone. If the skin condition at the puncture location is too thin to place the port, we can also tunnel down to select a suitable location. The probability of catheter kinking in the tunnelling technique is low, and there is a short distance even if the catheter is detached, so it will not fall into the blood vessel immediately. In clinical practice, we should consider the principle of good medical practice, trivializing our choice not only for aesthetic reasons, but also for the puncture site position.

In addition, the rates of complications including thrombosis, infections and skin exposure in this study were comparable to those of upper arm TIVAPs reported previously [1, 5, 17–20] (Table 2). There was no difference in the incidence of complications regardless of which vein (basilic, cephalic, brachial) was selected. We demonstrated a lower incidence of thrombosis rates with both incision techniques compared with other reports in this study (Table 2). There is a contradiction regarding whether the length of the catheter is connected to catheter-induced venous thrombosis [21, 22]. It is widely recognized that chemotherapy might increase the risk of thrombosis in patients with breast cancer [23, 24]. In this study, we have found that patients with hypertension have a higher rate of thrombosis. Patients with hypertension always experience slower peripheral blood flow and more severe vascular endothelial injury, which makes thrombosis easier to form. Anticoagulant medication should be started when catheter-related thrombosis is noticed. When anticoagulant therapy works, the port does not need to be removed. However, there is little evidence to suggest that prophylactic anticoagulation should be used often to avoid catheter-induced thrombosis, according to the literature [10].

Finally, previous literature reported that BMI was an independent risk factor for catheter-related infections [6]. In this study, we have explored a new result that patients with diabetes have a higher rate of infection. Diabetes is a significant risk factor for infection, so blood glucose levels should be strictly controlled to avoid infection. When choosing a puncture site incision, we should avoid placing the port directly below the incision, which may result in postoperative infection and other complications. Although the routine administration of prophylactic antibiotics has not yet been proposed in clinical practice, further studies are anticipated to explore whether prophylactic antibiotics can reduce the infection rate, especially in the context of diabetes. If it is suspected that the systemic infection is caused by the port, the catheter should be removed, and antibiotic therapy should be given [21]. Otherwise, port removal is not necessary when the infection is localized.

After years of use, totally implanted venous access ports in the upper arm are safe and convenient [17, 19]. They are also preferred due to their practicality and aesthetic outcomes. Female patients prefer the upper arm port because of the cosmetic results. When this puncture site incision is offered, the cosmetic result is better, and the operation time is shorter. Meanwhile, the rates of complications, including infection, thrombosis and skin exposure, in this study were comparable to those of traditional incisions. To the best of our knowledge, this is the first study on the novel technique of arm ports in breast cancer patients. It is important to note some restrictions. Since it is a retrospective single-centre design, the study may have been impacted by patient selection bias. A prospective clinical trial comparing the aesthetic effect and safety between two different incisions is anticipated.

## Conclusions

The findings of this study confirmed the feasibility and safety of TIVAPs in the upper arm with a novel incision technique in a large series of breast cancer patients. We believe that puncture site incision should be further pursued as a potential technique for improving aesthetic appearance and saving procedure time for patients requiring a port for breast cancer treatment.

## Data Availability

We declared that the materials described in the manuscript, including all relevant raw data, will be freely available to any scientist wishing to use them for non-commercial purposes, without breaching participant confidentiality.
